# The Twenty-First Anniversary of the Mississippi Valley Association of Dental Surgeons

**Published:** 1865-01

**Authors:** A. Berry


### THE TWENTY-FIRST ANNIVERSARY OF THE MISSISSIPPI VALLEY ASSOCIATION OF DENTAL SURGEONS.

"In compliance with a call from the Cincinnati Association of Surgeon Dentists, in connection with other members of the profession in the West, a convention of Professional Dentists met in Cincinnati, on Tuesday, the 13th of August, 1844," and organized the Mississippi Valley Association of Dental Surgeons.

The object of the society was to elevate the character and standing of the profession. Those of us who were present at its formation well remember the anticipations of good results from our associated efforts; and now, after the lapse of more than a score of years the retrospect is truly gratifying.

From the time of its organization to the present, the society has pursued the even tenor of its way in peace and harmony, and in no small degree promoted the object for which it was founded. Its discussions, and almost every subject connected with dental theory and practice, have ever been interesting and instructive; and its premiums for improvements in the manufacture of teeth instruments, essays, &c., have been liberal and promotive of the end in view.

As a means of elevating the standard of acquirements by which the profession should be entered, article 6 of the By-Laws reads thus: "Section 1, No member of this society shall take a student for a less time than two years, unless he shall have studied dentistry with some other reputable practitioner so as to make his term of pupilage equal to two years. It is provided however, that a graduate of any regular Medical College may be received for the term of one year. Section 2. Any member violating the preceeding section, shall be liable to impeachment and expulsion from this society."

In 1847 the society commenced the publication of the Dental Register of the West, which has for several years been the oldest dental journal in this or any other country.

This society about attaining its majority has been for several years the oldest dental association in existence. It is desirable that its future course, like its past be abundant in labors to promote the welfare of the profession and the interests of humanity.

In the onward march of the dental profession he who is isolated soon falls behind. Our profession, second only to the medical in alleviating and preventing human suffering, is so much benefited by interchange of views and discussions of societies, that probably no investment of time and money makes more profitable returns than that consequent on the attendence of the meetings of the Mississippi Valley Association of Dental Surgeons. Let every dentist in this section of the country, if possible, be present at the approaching anniversary, ready to aid in the common cause, and to enjoy the benefits resulting from associated effort.

A. BERRY.

